# Salivary Exosomal miRNA-1307-5p Predicts Disease Aggressiveness and Poor Prognosis in Oral Squamous Cell Carcinoma Patients

**DOI:** 10.3390/ijms231810639

**Published:** 2022-09-13

**Authors:** Aditi Patel, Shanaya Patel, Parina Patel, Dushyant Mandlik, Kaustubh Patel, Vivek Tanavde

**Affiliations:** 1 Biological and Life Sciences, School of Arts and Sciences, Ahmedabad University, Ahmedabad 380009, India; 2 Department of Head and Neck Oncology, HCG Cancer Centre, Ahmedabad 380060, India; 3 Bioinformatics Institute, Agency for Science Technology and Research (A*STAR), Singapore 138671, Singapore

**Keywords:** oral cancer, exosomes, saliva, microRNAs, prognosis, chemoresistance

## Abstract

**Background:** Salivary exosomal miRNAs as biomarkers facilitate repeated sampling, real-time disease monitoring and assessment of therapeutic response. This study identifies a single salivary exosomal miRNA prognosticator that will aid in improved patient outcome using a liquid biopsy approach. **Method:** Small RNA and transcriptome sequencing profiles of tumour tissues (*n* = 12) and salivary exosomes (*n* = 8) from oral cancer patients were compared to their non-cancerous counterparts. We validated these results using The Cancer Genome Atlas database and performing Real-time PCR on a large patient cohort (*n* = 19 tissue samples; *n* = 12 salivary exosomes). Potential target genes and the miRNA–mRNA networks and enriched biological pathways regulated by this microRNA were identified using computational tools. **Results:** Salivary exosomes (size: 30–50 nm) demonstrated a strong expression of CD47 and detectable expression of tetraspanins CD63, CD81 and CD9 by flow cytometry. miR-1307-5p was exclusively overexpressed in tissues and salivary exosomes of oral cancer patients compared to their non-cancerous counterparts. Enhanced expression of miR-1307-5p clinically correlated with poor patient survival, disease progression, aggressiveness and chemo-resistance. Transcriptome analysis suggested that miRNA-1307-5p could promote oral cancer progression by suppressing *THOP1*, *EHF*, *RNF4*, *GET4* and *RNF114*. **Conclusions:** Salivary exosomal miRNA-1307-5p is a potential prognosticator for predicting poor survival and poor patient outcome in oral cancers.

## 1. Introduction

Oral cancer is one of the most common subtypes of head and neck cancers, with high mortality and recurrence rates. Asia accounts for 48.7% of the total cases of oral squamous cell carcinoma (OSCC) reported globally [1]. Despite recent advances in treatment strategies, factors like metastasis, loco-regional invasion, and therapeutic refractoriness significantly influence the poor prognoses of patients and ultimately contribute to a low five-year overall survival rate. Determining the loco-regional aggression and the status of metastases during the initial staging may improve the overall survival and reduce the administration of aggressive treatments in the later course of the disease. The conventional approach for determining the aggressiveness of tumours is reliant on imaging technologies. The rates of regional, locoregional, and local recurrences in OSCC patients are 31.2–62.6%, 4.1–16.3%, and 24–51.1%, respectively; such recurrences ultimately require patients to undergo repetitive diagnostic processes [2]. Thus, it is necessary to understand the key factors driving OSCC prognoses and identify robust markers that may act as prognosticators for the disease.

Tumours shed exosomes and provide dynamic information about the tumour mass at the sample collection time point [3]. Emerging evidence suggests that tumour-derived exosomes are essential molecules that promote the formation of a pre-metastatic niche by transporting activators of epithelial-mesenchymal transition (EMT) (miRNAs, mRNAs, lncRNAs, etc.) to distant sites [4,5]. Circulating exosomal miRNAs (exomiRs) are packed into extracellular vesicles and are not prone to RNase-mediated degradation. Hence, they are more stable in various biofluids, like saliva, and are tissue-specific, thereby making them attractive biomarkers for cancer prognosis. Liquid biopsy is a non-invasive method which may facilitate repeated sampling and real-time monitoring of OSCC while allowing clinicians to observe the therapeutic response of patients. Saliva is the ideal biofluid for liquid biopsies in OSCC patients, as it exists in close proximity to the tumour. The potential of exomiRs as markers for disease progression, aggression, and risk prediction has been explored across various malignancies [6]. Previous studies have identified certain salivary exomiRs, like miR-486-5p, miR-134, miR-24-3p, and miR-200a, that can be used as markers for the early detection or screening of various oral cancers, including subsites in the tongue, pharynx, and hypopharynx [7,8,9]. However, the utility of salivary exomiRs as markers of OSCC progression, aggression, and prognosis is still unexplored. Furthermore, these studies have solely focused on the expression profiles of the aforementioned miRNAs. However, the underlying mechanisms responsible for tumour progression, as governed by these miRNAs, have not been explored.

The present study identifies miR-1307-5p as a novel candidate miRNA that is exclusively expressed in OSCC samples. Further, our data demonstrate that this miRNA may be a useful biomarker for predicting disease progression and prognosis. Additionally, we propose a mechanism via which this miRNA may regulate chemoresistance and the progression of OSCC.

## 2. Results

### 2.1. Identification and Characterisation of Salivary Exosomes Derived from Oral Cancer Patients

**Figure 1:**
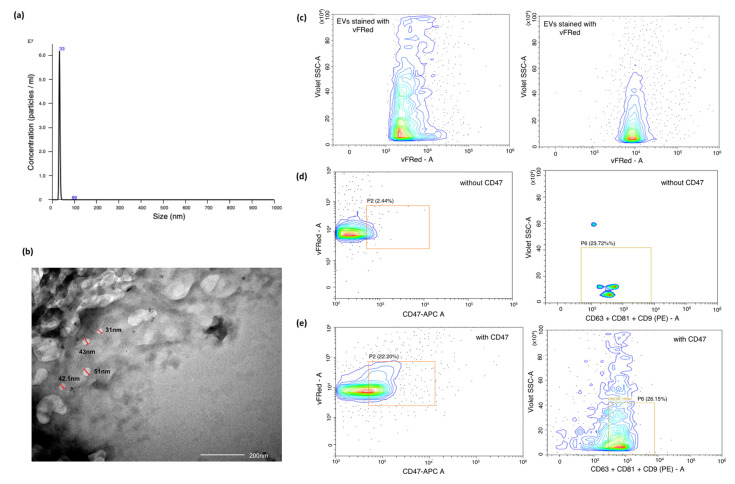
Characterisation of exosomes based on size, scatter and antigen expression. (**a**) Representative TEM images of exosomes from OSCC patients, (Scale: 200 nm); (**b**) Estimation of size and concentration of salivary exosomes from OSCC patients using Nanoparticle Tracking Analysis (NTA). The x-axis indicates the size distribution of particles and the y-axis shows the signal intensity in NTA; (**c**–**e**) Characterisation of exosomes by flow cytometry. We used a membrane-binding dye vFluor Red to stain exosomes and examined the violet side scatter v/s vFluor Red staining of these exosomes. This profile was similar to that of Lipo-50 liposomes used as a reference standard. Salivary exosomes were stained with antibodies against tetraspanins (CD63, CD81 and CD9) labelled with PE, and APC labelled CD47 antibody. We identified a distinct CD47+/vFluor Red+ population in the exosome population that was absent in the unstained control. This population was then examined for tetraspanin expression and showed detectable levels of tetraspanins. Since these particles stain with vFluor Red, have a violet side scatter similar to 50 nm liposomes, express CD47 and tetraspanins, we concluded that these are exosomes.

Exosomes were isolated from unstimulated saliva samples from patients diagnosed with OSCC and healthy individuals. The identification and characterisation of exosomes was focused on various criteria, such as size distribution and concentration using Nano-sight Tracking Analysis (NTA), validation of spherical morphology by Transmission Electron Microscopy (TEM) and the presence of exosome markers, as revealed by flow cytometry (Figure 1). 

NTA suggested that salivary exosomes appeared as a homogenous population with a mean size of 32.9 nm and a concentration of 3.66 × 10^8^ particles/mL (Figure 1a). Further TEM results revealed a sphere-shaped morphology of the vesicles with a size ranging from 40–50 nm, which was consistent with NTA profiles (Figure 1b). To assess the purity of the isolated exosomal population, the presence of CD47, CD63, CD9 and CD81 markers was estimated using flow-cytometric analysis. We initially calibrated the flow cytometer using 50 nm synthetic liposomes stained with vFluor Red, a dye that binds to lipid membrane. Salivary exosomes showed a similar violet-SSC and vFluor Red staining pattern to those of Lipo-50 synthetic liposomes, indicating a size around 50 nM. These exosomes showed a strong expression of CD47. We were also able to detect the expression of the tetraspanins CD9, CD63, and CD81, which are characteristic of exosomes (Figure 1c–e). Collectively, these findings indicated that the vesicles derived from the saliva of patients and healthy volunteers constituted a pure exosomal population. 

### 2.2. Small RNA Sequencing Analysis Identified miR-1307-5p, a miRNA Which Is Exclusively Expressed in Tissues and Salivary Exosomes of OSCC Patients

A preliminary focus of this study was to identify miRNAs which are exclusively expressed in tumours and salivary exosomes derived from OSCC patients with respect to their age-sex matched normal counterparts. We performed small RNA sequencing of the aforementioned cohort. In OSCC patient-derived tumour tissue and salivary exosomes, we observed a 260/280 ratio ranging from 1.87–2.12 (average 2.06) and an average RNA concentration of 655.02 ng/µL and 57.31 ng/µL, respectively. After pre-processing, adapter sequence trimming and filtering low quality reads, the mean q20 value for tissue-based libraries was 97.6%, whereas salivary exosome-derived libraries demonstrated a mean q20 value of 93.40%. An average of 25.4 million reads per sample were obtained for all tissue and salivary exosomes from OSCC patients, as well as from the normal counterparts. Reads shorter than 15 nucleotides and with a quality score < 30 were excluded to ensure the high quality of the sequencing results. Reads Per Million (RPM) was used to quantify miRNA expression level, with a false discovery rate (FDR) of ≤0.05, log2 fold change expression of ±2, and statistically significant abundance in each sample (*p* < 0.05).

**Figure 2:**
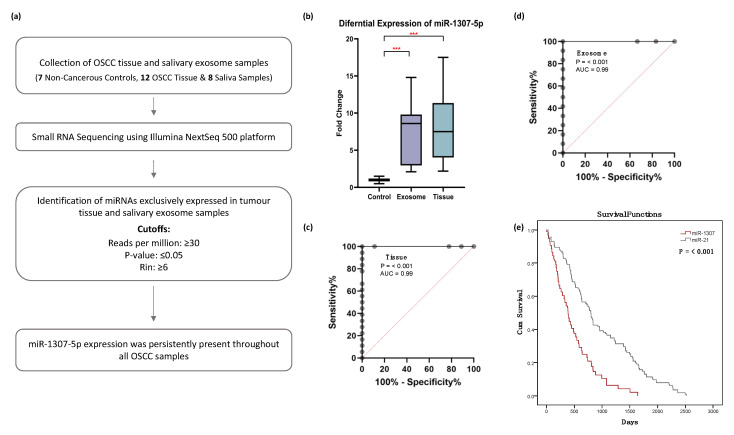
miR-1307-5p is differentially expressed and predicts poor overall survival in oral cancer patients (**a**) Data analysis pipeline for small RNA sequencing. (**b**) Box plots representing the expression of miR-1307-5p in 12 salivary exosomal samples of OSCC patients and 19 OSCC tissue samples compared to healthy controls. The expression levels of miRNAs were estimated using real-time PCR. The combined measure of sensitivity and specificity miR-1307-5p was 99.99% across (**c**) tissue and (**d**) exosome samples (AUC:0.99, 95 % CI, *p* < 0.001) Diagonal reference line acts as a performance measure of the diagnostic test. Note: AUC: Area Under the Curve, CI: Confidence Interval. (**e**) Kaplan–Meier plot of overall survival of miR-1307-5p and miR-21 generated from data available on TCGA_HNSC and data obtained from literature. The expression levels of miRNAs were estimated using real-time PCR. The data were normalised with U6 values, and the relative expression of miRNAs was analysed using the ddCt method. Buccal scrapings and saliva samples obtained from healthy controls were used to calculate the relative expression of miR-1307-5p in OSCC tissue and salivary exosomes respectively. Error bars represent mean ± SD of three independent experiments (*** *p* ≤ 0.001).

We identified 3 miRNAs that were significantly expressed in OSCC salivary exosome samples and 12 miRNAs with significant expression in OSCC tissue samples (Table A1). Out of these, only miR-1307-5p was found to be significantly upregulated in both tissue and salivary exosomal samples, with a log2 fold change >2 and an average read count of 493 in OSCC salivary exosomal samples and 1297 in OSCC tissue samples. Interestingly, the control samples reported an average read count of 23 in salivary exosomal and 47 in tissue samples, respectively. Given these low read counts in the controls, our results indicate that miR-1307-5p is significantly overexpressed in salivary exosomes and tumour tissues derived from OSCC patients compared to their non-cancerous counterparts (Figure 2a). Thus, enhanced expression of miR-1307-5p in the primary tumour could be reflected in the salivary exosomal fractions, making it a potential liquid biopsy-based biomarker for monitoring disease progression.

### 2.3. miR-1307-5p as a Potential Prognosticator in OSCC Patients 

Further, we substantiated the expression profile of miR-1307-5p in the validation cohort using Real-Time PCR. An upregulation of miR-1307-5p was observed in OSCC tissue (FC: 10.3 ± 7.9, *p*-value: 0.02) and salivary exosomal samples (FC: 7.3 ± 4.5, *p*-value: 0.0083), which was in agreement with the sequencing data (Figure 2b). 

The predictive power of miR-1307-5p was estimated using logistic regression models, in which miRNA expression profile was used as a predictor. Receiver operator characteristic (ROC) curves were determined, and area under the curve (AUC) was considered. The ROC values for miR-1307-5p showed statistically significant diagnostic strength of this miRNA, with a sensitivity and specificity of 99.99% across all sample types (AUC:0.99, 95 % CI, *p* < 0.001) (Figure 2c,d). 

Several reports have suggested that enhanced expression of miR-21 is associated with a poor overall survival rate in OSCC patients [10,11]. Hence, to ascertain whether miR-1307-5p exhibited potential as a prognosticator, we conducted a survival analysis of miR-21-5p with miR-1307-5p using their normalised expression value from TCGA datasets and a literature survey (205 OSCC patients). 

Our results suggested that miR-1307-5p could predict poor overall survival rate more effectively and significantly (50% of patients demonstrated mortality within 17 months) compared to miR-21-5p (*p*-value < 0.001) (Figure 2e). Collectively, these results suggest that miR-1307-5p offers predictive power regarding patient outcome and can efficiently function as a novel and independent prognostic biomarker for OSCC patients.

### 2.4. miR-1307-5p Demonstrates Clinical Association with Disease Progression, Aggressiveness, and Therapeutic Refractoriness

**Figure 3:**
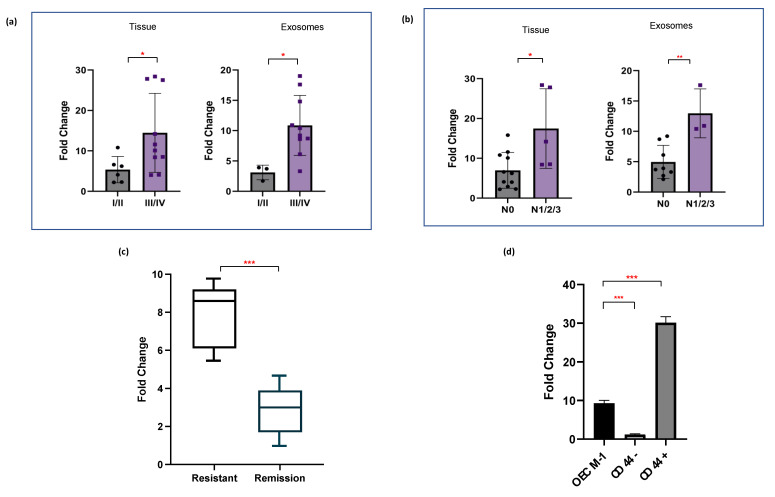
miR-1307-5p is overexpressed in late-stage tumours and refractory, chemoresistant tumours. Representative box plots depict relative expression patterns of miR-1307-5p in (**a**) early stages (Stage I/II) vs. late stages (Stage III/IV) of OSCC patient-derived tissue samples (*n* = 19) and salivary exosomal samples (*n* = 12), (**b**) OSCC tissue (*n* = 19) and salivary exosome (*n* = 12) samples of patients with no nodal metastasis (N0) vs those with metastasis (N1/2/3), and (**c**) salivary exosomal samples of OSCC patients with recurrent tumours and those with complete remission (*n* = 25). (**d**) Expression of miR-1307-5p in CD44+ and CD44− cells determined by qRT-PCR. The data were normalised with U6 values, and the relative miRNA levels were analysed using the ddCt method. Buccal scrapings and saliva samples obtained from healthy controls were used to calculate the relative expression of miR-1307-5p in OSCC tissue and salivary exosomes respectively. Expression of miR-1307-5p in OECM-1, CD44−, and CD44+ samples was compared to buccal scrapings obtained from healthy controls. Error bars represent mean ± SD of three independent experiments. An unpaired t-test was conducted to determine statistical significance (* *p* ≤ 0.05; ** *p* ≤ 0.01; *** *p* ≤ 0.001).

We further evaluated the relevance of enhanced miR-1307-5p expression with various clinicopathological parameters of OSCC patients. Increased levels of miR-1307-5p were observed in patients with high grade tumours (III/IV) (FC = 14.45 ± 9.76, *p*-value: 0.04) compared to low grade tumours (I/II) (FC = 5.37 ± 3.25). These findings were consistent in salivary exosomal samples of high grade (FC = 10.9 ± 5, *p*-value: 0.02) patients compared to low grade OSCC patients (FC = 3.1 ± 1.2) (Figure 3a). Moreover, elevated levels of miR-1307-5p were observed in patients with locoregional aggressiveness (N1, N2, N3) in both tumour tissues (FC: 17.5 ± 10, *p*-value: 0.03) and salivary exosomal samples (FC:13 ± 4, *p*-value: 0.0037) in comparison to those who did not report lymph node involvement (N0) (tissue FC = 7.5 ± 4.5; salivary exosome FC = 5 ± 2.7) (Figure 3b).

Further, to assess the significance of miR-1307-5p regarding real-time therapeutic monitoring, we analysed the expression levels of miR-1307-5p in chemoresistant OSCC patients compared to chemosensitive patients that showed complete disease remission post-treatment. A significant increase in the expression levels of salivary exosomal miR-1307-5p was observed in the chemoresistant cohort (FC: 4.82 ± 2.38, *p*-value: 0.01) compared to patients with complete remission (FC: 2.3 ± 1.2) (Figure 3c). Given that our analysis was conducted on a small patient cohort, further analysis is warranted. Considering that cancer stem cells (CSCs) are a group of quiescent cells within the tumour bulk that are believed to be responsible for tumorigenesis, resistance, relapse, and poor clinical outcomes, we evaluated the expression of miR-1307-5p in the CD44+ subpopulation derived from the OECM1 cell line [12]. CD44+ CSC were isolated from OECM1 using immunomagnetic bead separation. miR-1307-5p expression was significantly upregulated in the immuno-magnetically isolated CD44+ CSC subpopulation (FC: 31.3 ± 3.46, *p* = 0.0001) as compared to the CD44− (FC: 1.18 ± 0.16) fraction derived from the OECM1 cell line (Figure 3d). Collectively, these findings indicated that the presence of miR-1307-5p could be clinically associated with disease progression, local aggressiveness, and chemotherapeutic refractoriness, making it an ideal prognosticator for OSCC patients. Thus, it became imperative to understand its miRNA–mRNA networks and the underlying mechanism by which it regulates OSCC progression. 

### 2.5. Identification of Differentially Expressed Genes in OSCC Patients Using RNA Sequencing Analysis

An RNA-seq analysis of matched OSCC patients (that were utilised for small RNA sequencing) was conducted to identify mRNA targets and decipher the molecular and functional mechanism of miR-1307-5p. The simultaneous profiling of miRNAs and mRNAs provides an opportunity to compare the gene expression of miRNAs and their target mRNAs. This approach reduces the number of false positives typically associated with the miRNA-target prediction. A total of 17.8-28.5 million reads were obtained from tissue samples. Further, on an average, 62% of the reads were mapped with the reference human genome (hg38) with high confidence. A total of 10,660 differentially expressed mRNAs from tumour patient samples were identified. Upon comparing these with the TCGA datasets, we found that a total of 5868 significantly expressed mRNAs (log2FC = +2; *p* < 0.01) across these datasets, irrespective of the varying clinico-pathological status of the patients and discrepancies in the sequencing techniques. 

### 2.6. Target Gene Prediction and Functional Analysis of miR-1307-5p

**Figure 4:**
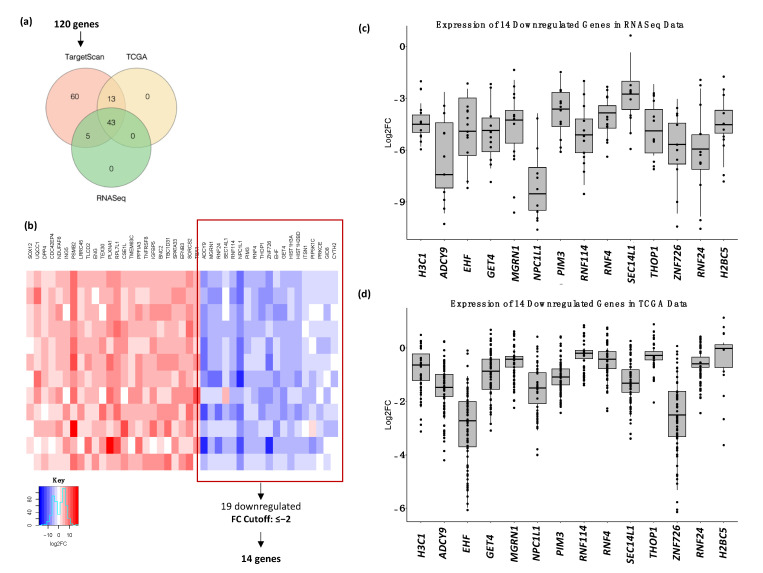
Validation of target genes of miR-1307-5p in OSCC. (**a**) Venn diagram depicting the selection of potential targets of miR-1307-5p via TargetScan 8, expression in TCGA_HNSC dataset and RNASeq expression generated in this study. 43 commonly expressed genes were identified out of which 30 were downregulated. A fold change cut-off of ≤−2 revealed 14 significantly downregulated genes. (**b**) Heatmap of the commonly expressed 43 genes showed 30 downregulated genes out of which 14 genes passed the FC cut-off of ≤−2. Representative boxplots show the expression of the shortlisted *HIST1H3A* (*H3C1*), *ADCY9*, *EHF*, *GET4*, *MGRN1*, *NPC1L1*, *PIM3*, *RNF114*, *RNF4*, *SEC14L1*, *THOP1*, *ZNF726*, *RNF24*, and *HIST1H2BD* (*H2BC5*) in (**c**) RNASeq and (**d**) TCGA_HNSC datasets.

**Figure 5:**
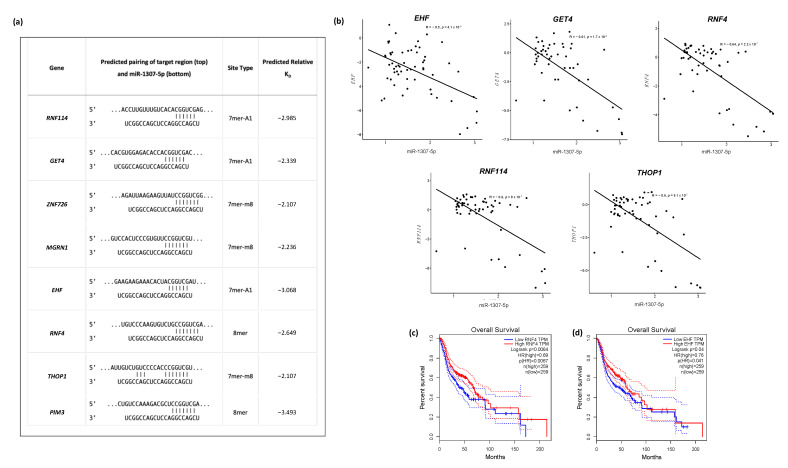
Predicted targets of miR-1307-5p show an inverse correlation with miR-1307-5p expression and predict overall survival in OSCC patients. (**a**) Table of predicted binding sites of target genes and miR-1307-5p, along with their respective predicted KD values; (**b**) Pearson’s correlation analysis using RNASeq expression data and the expression data in TCGA_HNSC to identify inversely correlated genes. Overall survival analyses were performed using the GEPIA platform where patients with (**c**) *RNF4* and (**d**) *EHF* expression above the median are indicated by red lines, and patients with gene expression below the median are indicated by black lines. Log-rank *p* < 0.05 was considered to indicate a statistically significant difference.

To identify the predicted target genes of miR-1307-5p, TargetScan was used. Out of the predicted 120 targets, 43 were significantly expressed across the TCGA_HNSC dataset and the data generated from RNASeq in this study (Figure 4a,b) (Table 1). Since miRNAs are believed to negatively regulate their targets, 14 downregulated genes (Figure 4c,d) were considered for further analysis [13,14]. A relative predicted KD cut-off of <−2 revealed eight genes with the highest binding affinity to miR-1307-5p: *THOP1*, *PIM3*, *MGRN1*, *EHF*, *RNF4*, *ZNF726*, *GET4*, and *RNF114*, with predicted relative KD values of −2.017, −3.493, −2.362, −3.068, −2.649, −2.107, −2.399, and −2.985, respectively (Figure 5a).

The regulation of gene expression by miRNA could result in an inverse correlation between the miRNA and its target [15]. To measure the inverse correlation between miR-1307-5p expression and the expression of the eight target genes, we performed Pearson correlation using RNA seq and TCGA HNSC expression data. Out of the eight identified genes, *THOP1* (*p* = 9.1 × 10^−7^), *EHF* (*p* = 4.1 × 10^−5^), *RNF4* (*p* = 2.2 × 10^−7^), *GET4* (*p* = 1.7 × 10^−6^), and *RNF114* (*p* = 8 × 10^−8^) showed significant correlation with the expression of miR-1307-5p (*p* < 0.0001) (Figure 5b). Amongst these, lower expression *RNF4* (*p* = 0.0064) (Figure 5c) and *EHF* (*p* = 0.04) (Figure 5d) correlated with poor overall survival in HNSCC patients in the TCGA datasets. 

## 3. Discussion

Exosomes have been intensively studied for disease progression, aggressiveness, and therapeutic monitoring in OSCC [16]. Moreover, the presence of exosomes in biological fluids such as saliva, cerebrospinal fluid, urine, and blood is providing a new source for biomarkers for OSCC [17,18]. However, until now, no standard method has been established for the isolation and characterisation of exosomes. Various surface markers such as CD9, CD63, CD81, and Alix have been identified as membrane protein markers for exosomes. Many reports have suggested that CD47 is also present on exosomes; however, whether CD47 plays a direct or an indirect role in determining exosomes remains unclear [19]. Our study showed significant CD47 expression in the salivary exosomes of OSCC patients, demonstrating the potential of individual exosome profiles in biomarker discovery. This is the first study to show the expression of CD47 on salivary exosomes; further research is required to understand its role as a probable surface marker for exosomes. 

The purpose of this study was to identify an exclusive biomarker for OSCC prognosis using salivary exosomal miRNAs. To this end, we conducted miRNA and transcriptome sequencing of salivary exosome and tissue samples from male patients with OSCC (subsite buccal mucosa) and a history of tobacco chewing. This study included only male patients, since OSCC in Western India is predominantly associated with the habit of chewing tobacco. This practice is much more prevalent among males than females. In our small patient cohort, all patients fitting our inclusion criteria were male. We found enhanced expression of miR-1307-5p in tumour tissues and exosomes as an independent risk predictor and prognosticator for OSCC patients. Concurring expression profiles of miR-1307-5p in salivary exosomes and tissue samples clearly suggested the crucial role and potential clinical utility of this non-invasive prognostic biomarker, using a liquid biopsy approach. This miRNA demonstrated a significant clinical association with disease progression, locoregional aggressiveness, and chemotherapeutic refractoriness. An abundance of miR-1307-5p in the CD44+ stem cell subpopulation derived from the OECM-1 cell line substantiated our clinical findings and was indicative of the fact that identified miRNA plays a role in regulating the self-renewal and maintenance of the CD44+ subpopulation that is responsible for disease aggressiveness and therapeutic resistance in OSCC patients. Few studies have reported the role of miR-1307-5p as an important prognosticator and risk predictor for various malignancies, specifically using non-invasive approaches. Xinyue Du (2020) reported the role of miR-1307-5p in proliferation and invasion by targeting *TRAF3* and activating the NF-κB/MAPK pathway in lung adenocarcinoma [20]. This study identified the miR-1307-5p/TRAF3/NF-κB/MAPK axis as a potential prognostic and therapeutic target. miR-1307 was found to be upregulated in chemoresistant ovarian tumour tissues and cell lines compared to the chemosensitive population, suggesting the role of miR-1307 in the development of chemoresistance in ovarian cancer [21]. Moreover, high levels of miR-1307 in the serum exosomes of ovarian cancer patients showed independent diagnostic power and were associated with tumour staging [22]. These results support our findings on the role of miR-1307-5p in disease progression, locoregional aggressiveness, and chemoresistance. However, the role of miR-1307-5p remains completely unexplored in head and neck/oral cancers. To the best of our knowledge, this is the first study to report the prognostic potential of miR-1307-5p in OSCC using liquid biopsies. 

Establishing the interrelationship of miR-1307-5p and its target genes would enhance our understanding of the relevant molecular mechanisms and provide potential therapeutic targets for oral cancers [23]. We identified 43 significantly expressed putative target genes of miR-1307-5p using transcriptome sequencing analysis, TCGA datasets, and the TargetScan software. Several studies have reported that miRNAs suppress gene expression by mRNA degradation and inhibiting protein translation or degrading the polypeptides through binding complementarity to 3′ UTR of the target mRNAs. Hence, in this study, we focused on the 14 downregulated target genes. Amongst these, five (*THOP1*, *EHF*, *RNF4*, *GET4*, *RNF114*) were found to have a significant association and formed strong miRNA–mRNA networks with miR-1307-5p based on significant expression patterns, alignment scores with the identified miRNA, and Pearson’s correlation scores using miRNA–mRNA expression patterns. 

EHF is a transcription factor that plays a vital role in cell differentiation and tumour-initiating and metastatic capability by conferring a CSC-like phenotype [24]. In HNSCC, the loss of EHF, either by point mutations or altered expression patterns, has been reported to increase the aggressiveness of the disease by inducing EMT phenomena or by targeting regulators of redox homeostasis, such as *NRF2* and *SOX2* [25,26]. These reports are in concordance with our findings and suggest a crucial role of EHF in promoting progression and aggressiveness in OSCC tumours.

In this study, among the top five genes targeted by miR-1307-5p, two belong to the E3 ubiquitin ligase family. Deregulated E3 ligases have been reported to confer uncontrolled proliferation, leading to malignant transformation, progression, and therapy resistance [27]. *RNF4* is associated with the double-strand break repair mechanism and is responsible for inducing the degradation of altered target proteins via proteasomes. *RNF4* has been reported to promote ubiquitination of PML bodies, which leads to proteasome degradation [28]. Moreover, deregulation of PML bodies leads to the activation of PPARδ mediated fatty acid oxidation pathways, which help in cancer stem cell maintenance, leading to disease progression and therapeutic refractoriness [29,30]. Another ligase, *RNF114*, has been reported to promote the ubiquitination and degradation of p21 [31]. p21 is an important regulator of the cell cycle; its stabilisation leads to cell cycle arrest and promotes apoptosis. This study demonstrated a significant downregulation of *RNF4* and *RNF114* in OSCC patients by miR-1307-5p. The suppression of these genes would likely not only impede apoptosis via cell cycle deregulation but would also promote cancer stem cell maintenance, leading to disease aggressiveness and therapeutic refractoriness. 

**Figure 6:**
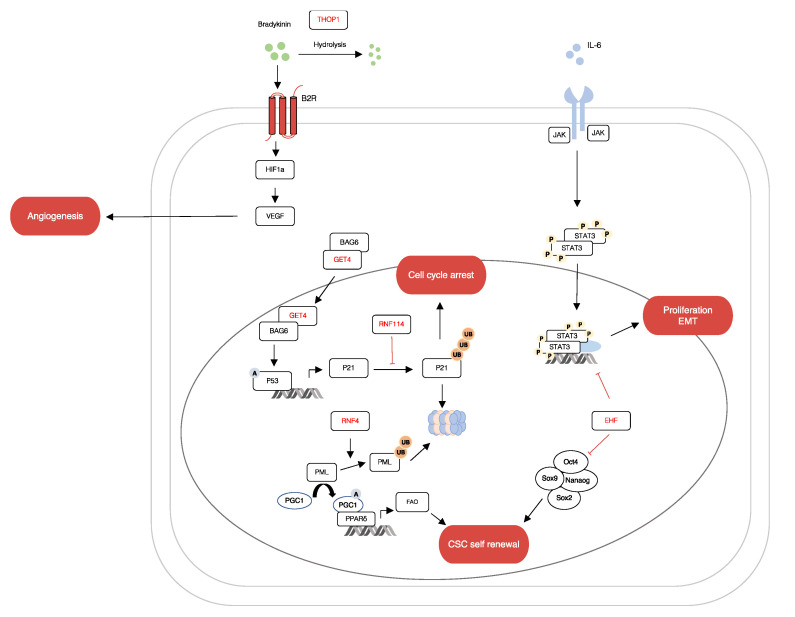
miR-1307 can regulate genes involved in OSCC progression. The figure shows a schematic representation of the relationships between putative genes targeted by miR-1307. Many of these genes modulate important cellular processes like cell proliferation, apoptosis, angiogenesis and maintenance of cancer stem cells.

*THOP1* is a characteristic metallopeptidase with a HEXXH zinc binding motif. It is associated with the metabolism of several neuropeptide acids, such as bradykinin, gonadotropin releasing hormone, opioids and neurotensin [32]. The hydrolysis of Bradykinin by *THOP1* induces the accumulation of intracellular calcium ions (Ca2+), leading to Ca2+ homeostasis which brings about tumour initiation, angiogenesis, progression and metastasis [33]. As such, further research on the degradation of neuropeptides by *THOP1* will lead to interesting models for future investigations into the development of cancer. Lastly, *GET4* is one of the factors of the BCL2-associated athanogene 6 (*BAG6*) chaperone complex. It functions as a regulator for the nucleo-cytoplasmic transport of *BAG6* [34]. In a recent study by Koike K et al., *GET4* was identified as a novel colorectal cancer driver gene that promoted tumour growth by facilitating cell cycle progression [35]. Collectively, these mRNA which are regulated by miR1307-5p modulate vital biological and functional processes such as cell cycle, proliferation, angiogenesis, EMT and CSC self-renewal (see Figure 6). Interestingly, to the best of our knowledge, apart from *EHF*, no studies have reported the association of *THOP1*, *RNF4*, *GET4* and *RNF114* with oral cancers. 

This study is a preliminary attempt to identify the role of miR-1307-5p as a potential prognosticator in OSCC using liquid biopsy techniques and to elucidate the mechanism by which this miRNA regulates mRNA targets (*THOP1*, *EHF*, *RNF4*, *GET4*, *RNF114*), thereby inducing disease aggressiveness and therapeutic refractoriness in OSCC patients. In order to better understand the efficacy of miR-1307-5p as a biomarker for OSCC, the findings of this study need to be validated in a larger and more diverse patient cohort. Additionally, a better understanding of the underlying functional role of the identified target genes and the regulatory mechanisms of miR-1307-5p will help us identify novel prognostic markers and therapeutic targets for oral cancers.

## 4. Materials and Methods

### 4.1. Patient Cohort and Sampling Details

**Figure A1:**
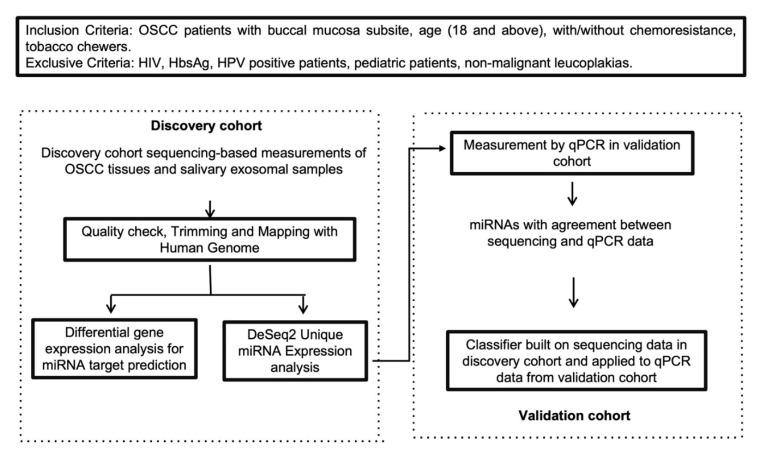
OSCC patient sample cohorts. Text without boxes denotes miRNA and mRNA sets. Boxes with solid lines denote analytical operations.

Samples of resected tumour tissue and unstimulated saliva were collected from patients diagnosed with oral squamous cell carcinoma (OSCC) (excluding tongue, larynx, pharynx and hypopharynx) from HCG Cancer Centre, Ahmedabad. Tissue samples were collected during the R0 resections of early- and late-stage OSCC patients, and tissue biopsies were taken from the tumour tissue. Resected tissue specimens were processed for histopathological evaluation, and additional tumour samples were snap-frozen and stored at −80 °C for further processing. Brush biopsies taken from healthy individuals with no etiological history of tobacco chewing and no clinically detectable oral lesions were used as normal controls. Whole saliva was collected into sterile tubes from healthy individuals and OSCC patients prior to their surgical resection according to the widely used protocol for the collection of saliva [36]. In order to ensure the collection of unstimulated saliva, the patients were instructed not to forcefully expectorate saliva. The saliva was then centrifuged at 2000× *g* for 10 min at room temperature to remove cells and debris. After transferring the supernatant into a sterile tube, exosome isolation using a precipitation method was performed (Figure A1).

**Figure A2:**
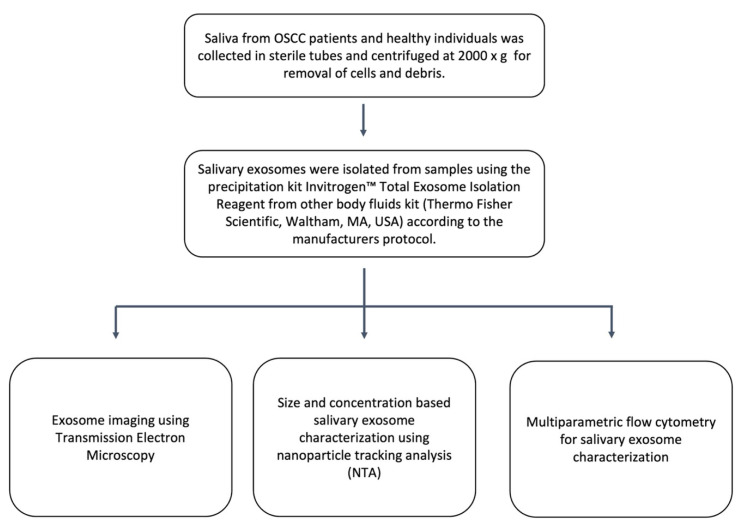
Exosome isolation and characterisation.

We divided the participants into a discovery cohort, consisting of four buccal scrapings (pooled) and three salivary exosomes from controls (pooled) and 12 OSCC tissue samples and eight salivary exosome samples from OSCC patients. We used pooled samples to generate a reference value for controls from multiple healthy volunteers. For buccal scrapings, we pooled four patients per sample, and for salivary exosomes, we pooled three volunteers per sample. The validation cohort consisted of five control buccal scrapings (pooled; three volunteers per pooled sample), five salivary exosomes from healthy volunteers, 19 OSCC tissue samples and 12 OSCC saliva samples. The characteristics of the patients included in the validation cohort were balanced for age and other clinicopathological factors. Additionally, both the discovery and validation cohorts included samples from male patients only (Figure A2). Demographic information and clinical data of the validation cohort is represented in Table 2. Patients diagnosed with oral squamous cell carcinoma, with buccal mucosa being the subsite of the primary malignancy and aetiology of smokeless tobacco, were included in the study. Patients with benign leucoplakia, HIV/HbsAg/HPV/COVID-19 infected patients, paediatric patients, patients whose sample may be required for repetitive diagnoses by the histopathologist or patients with disease-oriented complications were excluded from the study. Patients recommended by the oncologist under his supervision and consent were enrolled in this study.

Written informed consent for participation in this study was provided by all patients. This study was reviewed and approved by the HCG Cancer Centre’s Ethics Committee (ECR/92/Inst/GJ/2013/RR-16) for human subject research and the use of human tissues. It also complies with the guidelines set forth by the Declaration of Helsinki (2008).

To validate the findings of this study, miRNA-sequencing data from the TCGA-HNSC dataset (subsites: floor of mouth, gum, palate, and other and unspecified parts of the mouth) from the NIH National Cancer Institute Genomic Data Commons Data Portal (The Cancer Genome Atlas; https://cancergenome.nih.gov/, accessed on 1 May 2022) were downloaded. The miRNA sequencing results and clinicopathological and survival data of 114 oral cancer patients were included in our analysis. 

### 4.2. Exosome Isolation

Saliva of OSCC patients and healthy controls was diluted with PBS (1:1), followed by centrifugation at 2000× *g* for 10 min at room temperature. Exosomes were precipitated from the supernatant using Invitrogen™ Total Exosome Isolation Reagent from other body fluids kit (Thermo Fisher Scientific, Waltham, MA, USA), according to the manufacturer’s protocol. The precipitated exosomes were re-suspended either in TRIzol LS reagent (Thermo Fisher Scientific, Waltham, MA, USA) or in PBS, depending on the subsequent analysis to be performed.

### 4.3. Exosome Characterisation

The number of exosomes and their sizes were determined by Nanoparticle Tracking Analysis (NTA) on NanoSight LM10 (Malvern Panalytical Ltd., Malvern, UK) after diluting the exosome pellet in a physiologic solution (1:500). Imaging of negatively stained EVs (NanoVan, Nanoprobes, Yaphank, NY, USA) was carried out via transmission electron microscopy (TEM) using a Jeol JEM 1010 electron microscope (Jeol, Tokyo, Japan) (Figure A2). For imaging, exosomes were set onto 300 mesh carbon formvar grids followed by incubation with 5 μL 2% glutaraldehyde for 3 min. The excessive fluid was wick dried and the grids were rinsed with water. Samples were stained with 2% uranyl acetate for 1 min, followed by removal of excessive dye, and air-dried for 10 min. The samples were then examined at 100 kV on a transmission electron microscope at the Jeol Centre for Excellence in Imaging, Ahmedabad University.

Isolated exosomes were characterised by flow cytometry using the vFC EV Analysis kit (Cellarcus Biosciences, San Diego, CA, USA), according to manufacturer’s instructions. Briefly, samples were stained with a membrane stain (vFluor Red, Cellarcus Biosciences, CA, USA). The incorporation of a lipophilic membrane dye, i.e., vFluor Red, in the protocol helped us to discriminate between exosomes and electronic noise. The instrument was then calibrated using a vesicle size standard of synthetic 50-nm liposomes (Lipo50; Cellarcus Biosciences, CA, USA). Since the coincidence of events is a major challenge during the acquisition of exosomes, we measured the event rate during the acquisition of serially diluted samples. The lowest dilution where the event rate dropped in proportion with sample dilution was considered to be optimal, i.e., 1:80 for our salivary exosomal samples from OSCC patients. The samples (at optimal dilution) were then incubated with PE-tagged antibodies against tetraspanins (TS; CD9, CD63, and CD81) (Cellarcus Biosciences, CA, USA) and APC tagged CD47 antibody (Thermo Scientific, Waltham, MA, USA) for one hour at room temperature. Samples were acquired on the CytoFLEX-LX (Beckman Coulter, Brea, CA, USA) flow cytometer. Thresholding was set on the violet laser side scatter (v-SSC), and voltages were set according to the Cellarcus protocol. All samples were acquired using the high flow rate for 2 min. We used a hierarchical gating strategy to identify the exosomes expressing CD47 and tetraspanins. Since our exosomes expressed CD47 strongly, we used CD47 and vFluor Red as primary discriminators. Double positive events were then analysed for expression of tetraspanins, and events stained with vFluor Red, CD47 and tetraspanins were considered as exosomes. 

### 4.4. RNA Extraction

Exosomes: RNA from salivary exosomes was isolated using the method described by Prendergast et al. RNA was extracted by adding 750 μL TRIzol LS reagent (Thermo Fisher Scientific) and 200 μL chloroform to 40 μL exosomal sample [37]. We used 5 μL glycogen (5 mg/mL, Sigma Aldrich, St. Louis, MO, USA) instead of 3 μL, as described in the original protocol. The RNA pellet was resuspended in 32 μL of nuclease-free water. The final concentration of the purified RNA was determined using Qubit Fluorometer 4.

Tissues: TRIzol reagent was used to extract total RNA from OSCC tissue (as per the manufacturer’s protocol). The purity and yield of extracted RNA were determined using an Agilent 2100 Bioanalyzer (Agilent Technologies, Santa Clara, CA, USA) and Qubit Fluorometer 4 (Thermo Fisher Scientific, Waltham, MA, USA). 

### 4.5. miRNA Sequencing

The extracted total RNA from the samples was submitted for miRNA sequencing via the Illumina NextSeq500 platform. Small RNA library preparation and sequencing were performed by Genotypic Technology Pvt. Ltd., Bangalore, India. Small RNA libraries were prepared using the QIAseq^®^ miRNA Library Kit (Cat: 331502). The ligation of 3′ and 5′ adapters to the total RNA was followed by reverse transcription and amplification of the RNA using polymerase chain reaction (PCR), which resulted in the enrichment and barcoding of cDNA. Fragment size distribution of the libraries was followed by sequencing (12–15 million single end reads per run, per sample). FastQC was used to assess the overall quality of raw sequencing reads, and Cutadapt (v. 1.8) was used to trim the reads [38,39]. The filtered reads were mapped to the human reference genome (GRCh, version 38) using miRDeep2 (v0.0.7). The same toolkit was used to annotate potential novel miRNAs (cut-off: 6) and validate the predicted miRNAs from miRbase. DESeq2 was used to conduct statistical analyses of genes with read counts in ≥30 of the samples. A likelihood ratio test was conducted to contrast the conditions (leucoplakia-control)-(tumor-control) and obtain differentially expressed miRNAs among three levels (log2FC > 2; adjusted *p* < 0.01).

### 4.6. Transcriptome Sequencing

RNA extracted from tumour tissues and salivary exosomes of OSCC patients was subjected to mRNA-sequencing wherein library preparation and sequencing was performed by Genotypic Technology Pvt. Ltd., Bangalore, India. cDNA libraries were generated using Illumina TruSeq RNA Sample Preparation v2 Kit (Lot# RS-122-2001, RS-122-2002), followed by paired-end sequencing on the Illumina HiSeq 2500 (2x150bp). Raw sequencing reads were subjected to quality assessment using FastQC, followed by cleaning with Cutadapt. The refined reads were compared with a reference genome (GRCh, version 38) using Subread aligner.

### 4.7. miRNA Target Prediction

In order to identify the gene targets of miRNA, the TargetScan v8.0 prediction algorithm was used. Genes were considered differentially expressed if FC ≤ 2, and confidence was measured using predictive relative KD ≤ −2.

Pearson correlation and linear regression analysis were used to determine the correlation between the expression of miRNA and its target genes. For this, the RNASeq expression data were combined with the TCGA_HNSC expression data. The Pearson correlation coefficient was calculated in R, and the regression plots were made using the ggplot2 library in R.

### 4.8. Statistical Analysis

Receiver Operating Characteristic (ROC) curves were used to determine the discrimination power of the differentially expressed miRNAs as biomarkers for OSCC aggression and progression. GraphPad Prism 6.01 software was used to construct the curves. Then, the sensitivity, specificity, area under curve (AUC) and *p* value were calculated and the optimal threshold value was determined using Youden’s index (sensitivity + specificity − 1).

Survival analysis and t-tests to determine statistical significance were performed using SPSS software (v19.0; SPSS, Inc. Chicago, IL, USA) and GraphPad prism 9.0, respectively. The results are presented as mean ± standard deviation (SD) with a student’s t-test. All experiments were performed in triplicate and *p* < 0.05 was the threshold to determine statistical significance.

## Figures and Tables

**Table 1:** Expression of the predicted target genes of miR-1307-5p in TCGA dataset and the RNASeq data generated in this study.

Gene	Log2FC	
	TCGA	RNASeq
**Downregulated**		
*NPC1L1*	−6.24	−7.32
*ZNF726*	−3.05	−5.67
*HIST1H3A*	−4.89	−4.65
*ADCY9*	−4.23	−3.98
*EHF*	−2.51	−3.06
*RNF4*	−2.52	−2.89
*HIST1H2BD*	−2.95	−2.74
*RNF24*	−2.67	−2.45
*GET4*	−2.41	−2.41
*PIM3*	−2.77	−2.34
*GID8*	−1.68	−2.27
*THOP1*	−2.27	−2.19
*RNF114*	−2.65	−2.02
*MGRN1*	−2.29	−1.95
*SEC14L1*	−2.34	−1.82
*CYTH2*	−1.18	−1.72
*PIP5K1C*	−1.98	−1.50
*ITSN1*	−2.36	−1.29
*PRKCE*	−1.93	−0.88
**Upregulated**		
*SOX12*	1.56	2.19
*UQCC1*	2.17	2.46
*ENG*	3.54	2.79
*NDUFAF6*	2.95	3.30
*TLCD2*	3.50	3.31
*ING5*	3.03	3.37
*LRRC45*	3.22	3.40
*DPP4*	2.60	3.63
*CDC42EP4*	2.79	3.63
*PSMB2*	3.19	3.90
*CSE1L*	3.94	3.97
*TEX30*	3.56	4.05
*RPL7L1*	3.86	4.06
*TMEM63C*	4.18	4.11
*PPFIA3*	4.83	4.72
*PLXNA1*	3.86	4.73
*IGFBP5*	7.18	5.33
*TNFRSF8*	6.07	6.78
*SPATA33*	7.92	8.27
*EFNB3*	8.48	8.50
*BNC2*	7.29	8.56
*TBC1D31*	7.77	8.98
*SORCS2*	8.78	9.54

**Table 2:** Clinicopathological characteristics of OSCC patients included in the validation cohort.

Characteristic	Value = N%
**Gender**	
Male	21 (100%)
**Sample Type**	
Tissue	19
Saliva	12
**Age group at diagnosis (years)**	
<50	11 (52.4%)
>50	10 (47.6%)
Age at diagnosis (years, M ± SD)	48 ± 83
**Stage**	
I/II	8 (39.1%)
III/IV	13 (61.9%)
**Lymphovascular Invasion**	
N0	15 (71.4%)
N1	0
N2	2 (9.5%)
N3	4 (19.1%)
**Relapse**	
Yes	3 (14.3%)
No	18 (85.7%)

## Data Availability

The data will be available on request to the corresponding author.

## Appendix A

**Table A1:** List of miRNAs expressed exclusively in OSCC salivary exosome samples and tissue samples.

Unique miRNAs in OSCC Salivary Exosome Samples	Unique miRNAs in OSCC Tissue Samples
hsa-miR-125a-5p	hsa-miR-10b-5p
**hsa-miR-1307-5p**	hsa-miR-128-1-5p
hsa-miR-330-5p	hsa-miR-130a-5p
	hsa-miR-138-5p
	hsa-miR-139-5p
	hsa-miR-146b-5p
	hsa-miR-214-5p
	hsa-miR-365b-5p
	hsa-miR-382-5p
	hsa-miR-409-5p
	hsa-miR-671-5p
	**hsa-miR-1307-5p**
